# Characteristics of Rare Inherited Retinal Dystrophies in Adaptive Optics—A Study on 53 Eyes

**DOI:** 10.3390/diagnostics13152472

**Published:** 2023-07-25

**Authors:** Katarzyna Samelska, Jacek Paweł Szaflik, Maria Guszkowska, Anna Katarzyna Kurowska, Anna Zaleska-Żmijewska

**Affiliations:** 1 Department of Ophthalmology, Medical University of Warsaw, 02-091 Warsaw, Poland; 2 SPKSO Ophthalmic University Hospital, 00-576 Warsaw, Poland

**Keywords:** adaptive optics, cone dystrophy, cone-rod dystrophy, inherited retinal diseases, inherited retinal dystrophies, photoreceptors, retinal imaging, Stargardt disease

## Abstract

Inherited retinal dystrophies (IRDs) are genetic disorders that lead to the bilateral degeneration of the retina, causing irreversible vision loss. These conditions often manifest during the first and second decades of life, and their primary symptoms can be non-specific. Diagnostic processes encompass assessments of best-corrected visual acuity, fundoscopy, optical coherence tomography, fundus autofluorescence, fluorescein angiography, electrophysiological tests, and genetic testing. This study focuses on the application of adaptive optics (AO), a non-invasive retinal examination, for the assessment of patients with IRDs. AO facilitates the high-quality, detailed observation of retinal photoreceptor structures (cones and rods) and enables the quantitative analysis of parameters such as cone density (DM), cone spacing (SM), cone regularity (REG), and Voronoi analysis (N%6). AO examinations were conducted on eyes diagnosed with Stargardt disease (STGD, N=36), cone dystrophy (CD, N=9), and cone-rod dystrophy (CRD, N=8), and on healthy eyes (N=14). There were significant differences in the DM, SM, REG, and N%6 parameters between the healthy and IRD-affected eyes (p<0.001 for DM, SM, and REG; p=0.008 for N%6). The mean DM in the CD, CRD, and STGD groups was 8900.39/mm${}^{2}$, 9296.32/mm${}^{2}$, and 16,209.66/mm${}^{2}$, respectively, with a significant inter-group difference (p=0.006). The mean SM in the CD, CRD, and STGD groups was 12.37 μm, 14.82 μm, and 9.65 μm, respectively, with a significant difference observed between groups (p=0.002). However, no significant difference was found in REG and N%6 among the CD, CRD, and STGD groups. Significant differences were found in SM and DM between CD and STGD (p=0.014 for SM; p=0.003 for DM) and between CRD and STGD (p=0.027 for SM; p=0.003 for DM). Our findings suggest that AO holds significant potential as an impactful diagnostic tool for IRDs.

## 1. Introduction

Inherited retinal dystrophies (IRDs), also known as inherited retinal diseases, are genetic disorders characterized by a variety of inheritance patterns, all leading to bilateral, irreversible vision loss. Recognized as rare diseases, the IRD group is highly heterogeneous, comprising over 20 phenotypically distinct conditions. Each condition may arise from different mutations in distinct genes. To date, around 271 genes have been linked with IRDs. The disease progression may vary among patients, with functional blindness occurring at different ages. The genetic basis of the IRDs of different phenotypes is detailed on RetNet (https://web.sph.uth.edu/RetNet/home.htm (accessed on 9 July 2023). The genetic characteristics of patients with IRDs have been described in various populations, such as in Taiwan [1]. Only one successful therapy has been introduced so far, voretigene neparvovec-rzyl—Luxturna™—limited to patients with mutations in the *RPE65* gene. Other treatments, including stem cell therapy, retinal pigment transplantation, photoreceptor transplantation, and anti-inflammatory approaches, are under development [2,3,4,5,6,7,8]. However, none of these therapies have seen widespread use.

### 1.1. Retinitis Pigmentosa

Retinitis pigmentosa (RP) is the most common IRD, with a prevalence of 1/3000 to 1/5000, accounting for approximately half of all IRDs. RP is a rod-cone dystrophy (RCD) in which the deterioration of rod function exceeds that of cone function, leading to the loss of photoreceptor and pigment epithelium function. RP’s heterogeneous origin involves more than 3100 mutations in over 50 genes, including the non-syndromic form. The disease may be inherited in an autosomal (dominant or recessive) or X-linked pattern, with mitochondrial inheritance uncommon in the non-syndromic form [9,10]. The age of onset varies among patients with different forms of the disease, with RP potentially affecting visual function in early infancy, as well as in the second to third decades of life. Retinitis pigmentosa presents heterogeneous genetics, clinical phenotypes, and presentations. A single gene mutation may lead to different clinical presentations, even among members of the same family [10].

RP may manifest as an independent disease or as part of a syndrome, such as Usher syndrome, which includes hearing loss, or Bardet-Biedl syndrome, which features RP accompanied by kidney failure, polydactyly, and obesity. These syndromic forms are also genetically heterogeneous: there are at least 12 gene mutations leading to Usher syndrome and at least 17 gene mutations causing Bardet-Biedl syndrome [10].

A crucial symptom of RP is the narrowing of the visual field, which occurs in the advanced stages of the disease. Since the degeneration primarily affects the peripheral retina, the central vision is not impacted in the early stages, and potential findings include nyctalopia and photophobia. The clinical image is characterized by peripheral bone spicule deposits, attenuation of the retinal blood vessels, optic disc pallor, and, in later stages, the development of macular edema. Some RP patients develop subcortical posterior cataracts [11].

Leber congenital amaurosis (LCA) is a form of RP present in infancy, resulting in vision loss in early infancy. One of the genes that may be affected in LCA is *RPE65*. Mutations in this gene usually cause the autosomal recessive form of the disease but can also lead to the dominant form [10]. LCA caused by *RPE65* mutation may be treated with voretigene neparvovec-rzyl (Luxturna™). Luxturna™ is the first commercially available gene therapy, approved by the FDA in December 2017 and now globally available [12,13].

The cone spacing in RP has been found to be increased compared to healthy eyes, correlating with microperymetry changes [14].

Despite RP being the most common condition among IRDs, we chose not to include RP patients in our study for two reasons. Firstly, in retinitis pigmentosa, the morphological changes primarily affect the rods, which are challenging to quantitatively visualize with adaptive optics. Secondly, the morphological changes predominantly affect the peripheral retina; hence, we opted for conditions primarily affecting the macular region.

### 1.2. Characteristics of Stargardt Disease (STGD), Cone Dystrophy (CD), and Cone-Rod Dystrophy (CRD)

#### 1.2.1. Stargardt Disease (STGD)

**Figure 1:**
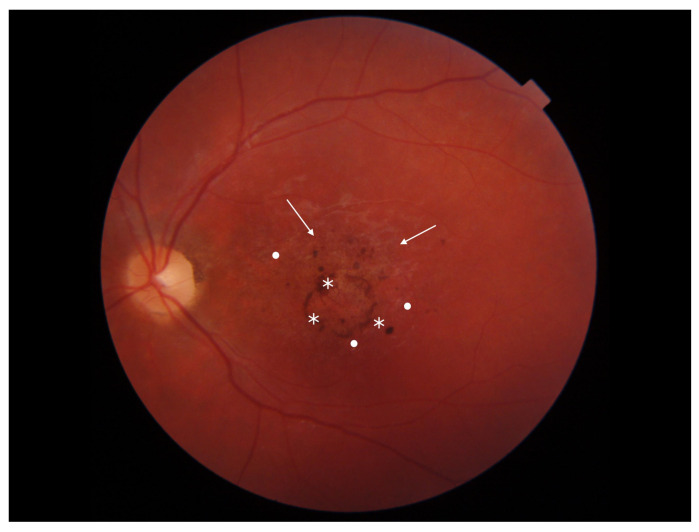
Eye fundus image of a patient with Stargardt disease (DRI OCT Triton; Topcon). Observe the ‘bull’s eye’ maculopathy (indicated by arrows), pigment deposits (indicated by asterisks), and the presence of yellow-white flecks (highlighted with dots) in the perifoveal area.

Stargardt disease (STGD) is the most common maculopathy among IRDs, with a prevalence of approximately 1 in 10,000. STGD is characterized by central vision loss, dyschromatopsia, and macular abnormalities, often forming a ‘bull’s eye’ sign. Yellow-white flecks in fundus autofluorescence (FAF) indicate abnormal lipofuscin accumulation within the retinal pigment epithelium (RPE). An eye fundus image of STGD is presented in Figure 1.

Typically, symptoms of the disease begin in the second decade of life. Maculopathy arises from abnormal lipofuscin deposits in the central macula. These deposits block the signal from the underlying choroid, causing the characteristic ‘dark choroid’ sign in fluorescein angiography (FFA) [15,16].

Diagnosis is based on clinical signs and can be confirmed by genetic testing. The most common mutations in Stargardt’s disease affect the *ABCA4* gene (MIM601691), which codes for a protein located in the outer segments of the photoreceptors [17]. Disease monitoring includes functional tests, such as best-corrected visual acuity (BCVA) assessment, multifocal electroretinography (mfERG), and microperimetry, and imaging tests such as spectral-domain optical coherent tomography (SD-OCT), FAF, and FFA [16,18,19,20].

Disease progression can be determined by tracking the ellipsoid zone loss in SD-OCT [21] and the rate of atrophy enlargement (RAE) monitored in FAF [22,23,24].

AO imaging (both spectral and confocal) has been utilized to assess photoreceptor abnormalities in Stargardt disease. Studies have found increased cone spacing (SM) and decreased cone density (DM) compared to the normal retina [18,21,25,26]. Cone reflectivity changes have been described but do not correspond with lipofuscin accumulation [25]. Moreover, confocal AO images show changes in retinal photoreceptor morphology, detectable even before OCT and FAF can identify changes. These images reveal ‘dark spaces’ within photoreceptor structures. These ‘dark spaces’ indicate abnormal cones with decreased core reflectance and a disrupted outer segment structure, although their inner structure appears intact. These ‘dark spaces’ could potentially be a target for treatment. It is worth noting that ‘dark spaces’ are not pathognomonic for STGD and can also be found in other IRDs [26,27,28]. Adaptive optics studies have suggested that photoreceptor loss in patients with type STGD1 Stargardt disease precedes clinically detectable retinal pigment epithelial disease [26]. Conversely, the highly reflective structures observed in AO might represent flecks or areas of atrophy [29].

An additional study analyzing the AO visualization of Stargardt disease illustrated differences in cone morphology between the perifoveal area, the transition zone, and the outer retina [30]. In macular atrophy regions, the RPE mosaic was not clearly identifiable in two of the three patients, and the photoreceptors were unidentifiable in the remaining patient. In transition regions, the cones appeared dark, enlarged, and sparse, and the cone spacing was increased. The AO image of the peripheral retina showed cone spacing and an appearance similar to those of a healthy retina, but the RPE cell contrast was lower than in normal eyes.

**Figure 2:**
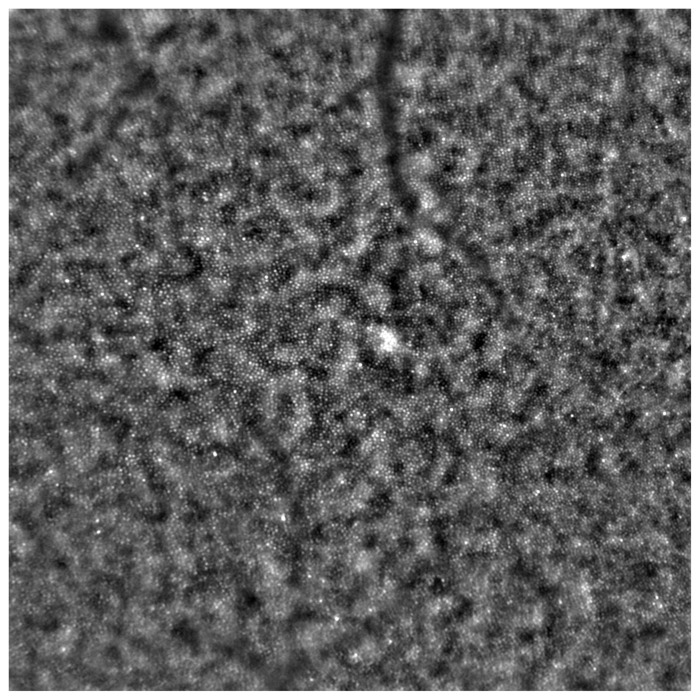
Adaptive optics image showcasing the photoreceptors of a healthy eye (Rtx1™; Imagine Eyes, France). Individual cones are distinctly visualized (visible as white and grayish dots), and the cone mosaic image appears undisrupted.

**Figure 3:**
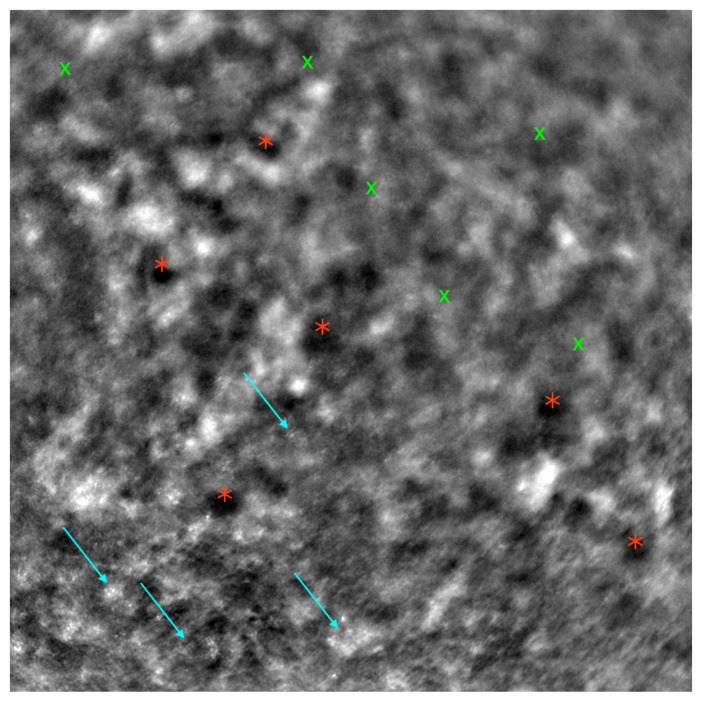
Adaptive optics image demonstrating the photoreceptors of an eye with Stargardt disease (Rtx1™; Imagine Eyes, France). Observe the disruption in the cone mosaic (examples indicated by light blue arrows) and the presence of ‘dark spaces’ (examples highlighted with red asterisks). The area with inadequate visualization of the cone mosaic is marked with green X symbols.

Figure 2 depicts an AO image of a healthy retina, and Figure 3 presents an AO image of an STGD retina.

Among other IRDs, cone-rod dystrophy (CRD) and cone dystrophy (CD) are notable for their primary cone dysfunction, which supersedes rod dysfunction. In these disorders, the macula, being the retinal region with the highest cone concentration, is primarily affected.

#### 1.2.2. Cone-Rod Dystrophy and Cone Dystrophy

**Figure 4:**
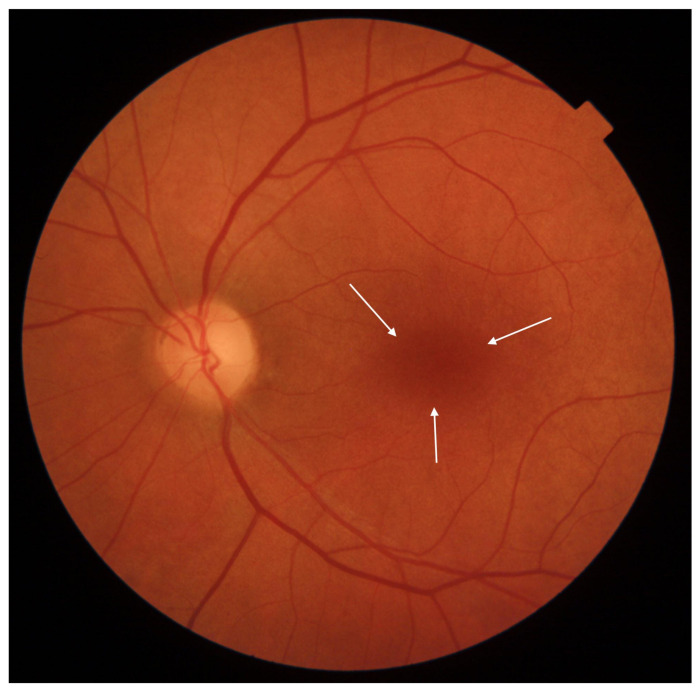
Eye fundus image of a macula with cone dystrophy (DRI OCT Triton; Topcon). Observe the ‘bull’s eye’ maculopathy (indicated by arrows).

**Figure 5:**
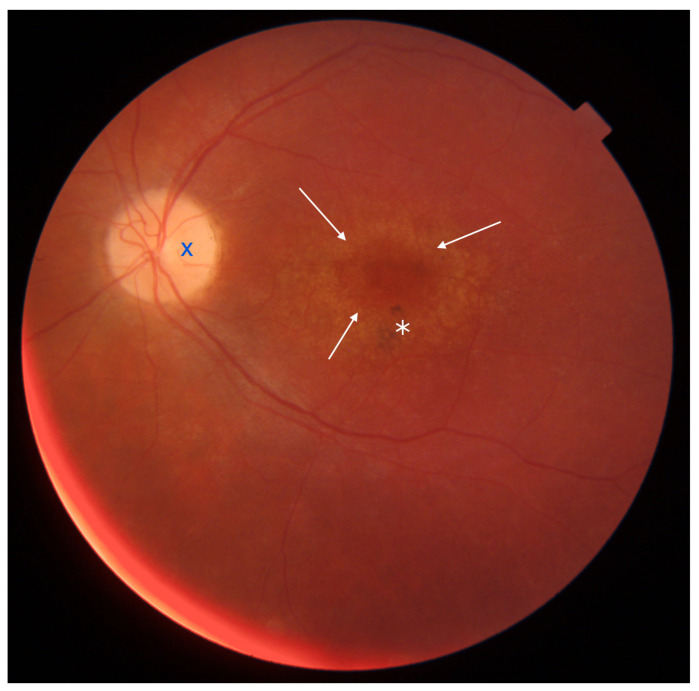
Eye fundus image of a macula with cone-rod dystrophy (DRI OCT Triton; Topcon). Note the ‘bull’s eye’ maculopathy (indicated by arrows), pigment deposits in the perifoveal area (marked with an asterisk), and the pallor of the optic nerve disc (marked with dark blue X symbol).

Cone-rod dystrophy (CRD) and cone dystrophy (CD) are IRDs that occur less frequently than Stargardt disease. CRD involves both cones and rods, while CD affects only cones. Both primarily impact the macula, leading to central vision disturbances. The clinical presentation is similar in both CD and CRD, showcasing a bull’s eye maculopathy and bone spicule deposits in the outer retina. Inheritance patterns can be autosomal dominant, autosomal recessive, X-linked, or unresolved. At least 32 gene mutations associated with these dystrophies have been identified. Symptoms of CD encompass central vision deterioration, nystagmus, photophobia, and color vision deficits [31,32]. Early nyctalopia is not a feature of CD [32]. CRD symptoms appear later than CD symptoms and often result in legal blindness in later life [33]. Fundus photographs of eyes with CD and CRD are shown in Figure 4 and Figure 5, respectively.

**Figure 6:**
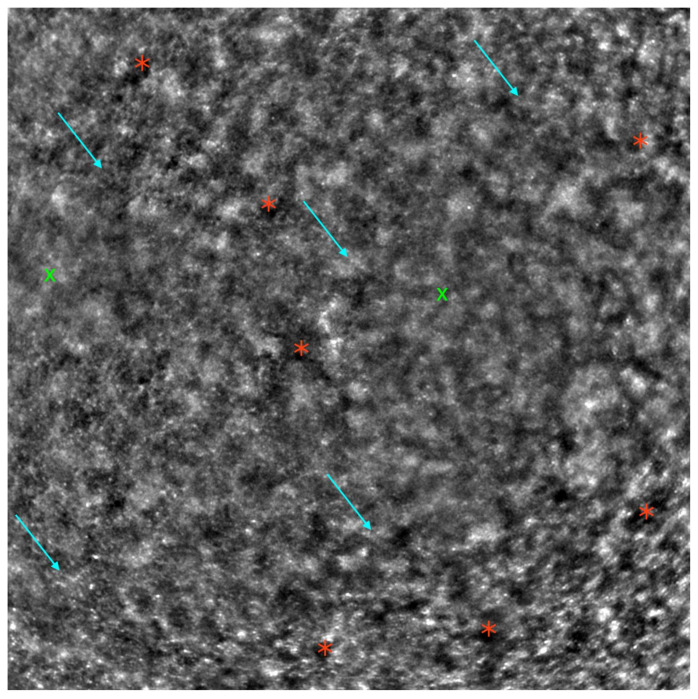
Adaptive optics image of the photoreceptors in an eye with cone dystrophy (Rtx1™; Imagine Eyes, France). Observe the disruption of the cone mosaic (examples indicated by light blue arrows) and the presence of ‘dark spaces’ (examples indicated by red asterisks). The areas with poor visualization of the cone mosaic are marked with green X symbols.

**Figure 7:**
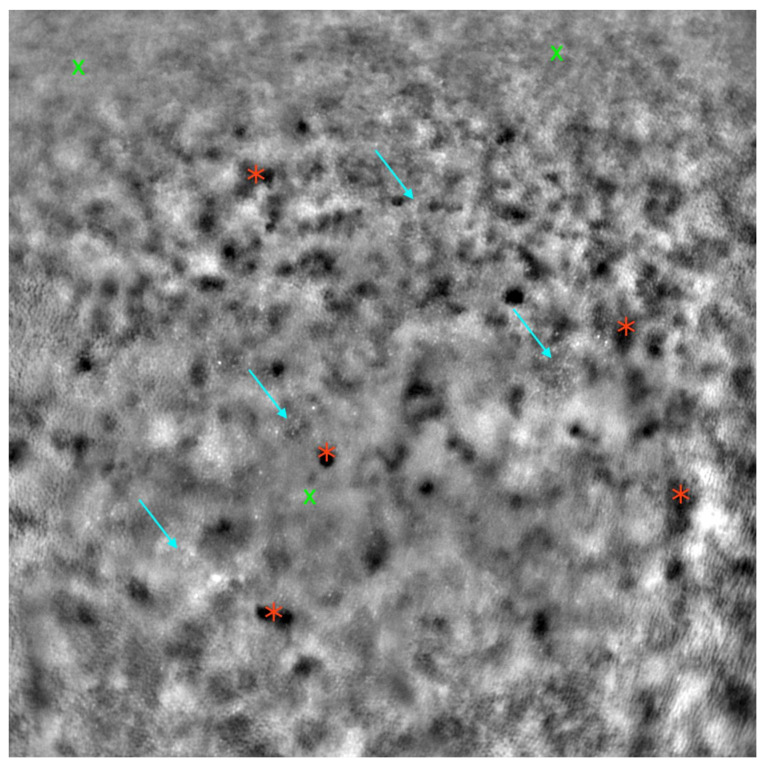
Adaptive optics image of the photoreceptors in an eye with cone-rod dystrophy (Rtx1™; Imagine Eyes, France). Observe the disruption of the cone mosaic (examples indicated by light blue arrows) and the presence of ‘dark spaces’ (examples indicated by red asterisks). Areas with poor visualization of the cone mosaic are marked with green X symbols.

The characteristic AO imaging pattern in CRD has been described as areas devoid of cones, although it has also been reported as a diminished cone mosaic with reduced cone density and increased cone spacing [34,35,36]. Other studies have noted that eyes with CRD exhibit increased cone spacing compared to healthy eyes and those with RP. Correlations between CRD and the deterioration of mfERG peak amplitudes have also been reported [37]. An AO scanning laser ophthalmoscopy (AOSLO) study highlighted greater cone loss than rod loss, reflecting the disease’s nature [38]. AO images from CD and CRD cases are presented in Figure 6 and Figure 7.

A study on AO imaging in cone dystrophy with supernormal rod electroretinogram (CDSR) depicted reduced cone density, disruption of the cone photoreceptor mosaic, and the presence of cones surrounded by patches of either absent or present non-wave-guiding cones [31].

Cone-rod dystrophy, cone dystrophy, and Stargardt disease primarily affect the macula and central visual field, resulting in progressive central vision loss. This differs from RCDs, which initially impact peripheral vision. The clinical implications of central vision loss are significant, as patients and their families must confront the possibility of progressive vision deterioration.

Retinal imaging, designed to assess the stage and monitor the progression of maculopathies, is achievable using OCT, AF, FAF, electrophysiological testing (such as mfERG), and perimetry (such as 10-2 macular perimetry).

However, monitoring with the above-mentioned methods can often prove challenging and imprecise. As a more recent imaging technique, AO allows the visualization of the retina’s microstructures, such as the cones, expanding the diagnostic and monitoring capabilities for IRDs. Studies confirm that morphological changes in the cone microstructure, as visualized by AO, can be detected earlier than the onset of functional visual deterioration [39].

#### 1.2.3. Adaptive Optics

Adaptive optics (AO) is an imaging technique originally developed for precise visualization in astronomy, capable of correcting atmospheric irregularities. This aberration-correcting system provided high-quality imaging of distant objects [40,41]. In 1989, Dreher et al. [42] developed improvements in laser scanning tomography, enabling the compensation of corneal and lens aberrations to assess the retinal nerve fiber layer (RNFL) thickness and optic disc topography of the human eye. It was not until 1997, however, that Liang et al. first utilized adaptive optics to visualize single cells in the human retina [43].

The retinal imaging AO camera uses two light sources: one to illuminate the retina and the second to measure and correct wavefront aberrations. The wavefront sensor and corrector measure these aberrations. The control system then interprets the data collected by the sensor and orchestrates the interaction between the sensor and corrector [41,44]. There are two main AO technologies used in visualizing retinal photoreceptors: split detector (SD-AOSLO) and confocal (cSLO). Both confocal and spectral images can be taken simultaneously. Some AO imaging devices capture three channels simultaneously (confocal, split detection, and dark-field), each highlighting different retinal structures [18,44,45].

AO permits the visualization of rods and cones, the two types of retinal photoreceptors. AO can measure parameters such as cone density, cone spacing, Voronoi analysis, reflectivity, regularity, the preferred orientation of cones, and local spatial anisotropy [18,46,47,48]. While rod visualization is possible in healthy individuals, determining their spacing is generally impractical. However, in retinas with a disrupted cone mosaic, it can reveal the presence of often-enlarged rods. The cone-to-rod ratio can be measured [26,27]. For our project, we opted not to take rod measurements.

Beyond photoreceptor visualization, AO also provides images of the retinal microvasculature. Studies examining the arteriole morphology in angiopathies, such as diabetic retinopathy, and in systemic conditions such as hypertension, obesity, and prediabetes, offer an innovative perspective on retinal blood circulation [49,50,51,52,53].

AO’s capability to visualize individual photoreceptors due to aberration correction is a breakthrough in modern ophthalmic imaging. Research on its application in IRDs is emerging, as existing diagnostic techniques (SD-OCT, FAF, FA, and microperimetry) are not sufficiently precise for comprehensive assessments. Several studies confirm that IRDs can present morphological changes detectable by AO preceding functional vision loss, which could greatly impact IRDs’ diagnostic processes [36,39,44].

The evaluation of RNFL and optic nerve microvasculature microchanges in glaucoma has also been a subject of study. Age-related macular degeneration (AMD), a condition causing morphological changes in the macula, has been a focus in numerous studies. AMD, along with diabetic retinopathy and glaucoma, remains one of the leading causes of vision loss in developed countries [44,54].

While there are numerous studies examining AO images in IRDs, there is still much to discover and describe. AO has been in use in ophthalmology for over two decades, and there is still a need for longitudinal studies monitoring cone density in IRDs [55].

#### 1.2.4. Rtx1™

Rtx1™ (Imagine Eyes, Orsay, France) is an adaptive optics flood illuminated ophthalmoscope (AOFIO) that utilizes infrared light with a wavelength of 850 nm and offers a resolution of 1.6 μm. The image dimensions that it captures are 4${}^{\circ }$ × 4${}^{\circ }$, corresponding approximately to 1.2 mm × 1.2 mm of the retina. The location under examination can be selected (for example, 2${}^{\circ }$ superior, inferior, temporal, or nasal). Image acquisition in a single position takes between 2 and 4 s, during which 40 individual images are acquired [44,49,53,56]. The Rtx1™ software (*version 3.4*, also called *AO Image 3.4*, Imagine Eyes, Orsay, France) includes two programs for data evaluation: AO Detect for photoreceptor parameter analysis and AO Detect Artery for vessel parameter analysis.

## 2. Materials and Methods

This study focused on examining the characteristic images and parameters of the macular cones in three conditions predominantly affecting the posterior pole, STGD, CRD, and CD, using AO device Rtx1™.

The investigation focused on the photoreceptor parameters, namely cone density (DM), cone spacing (SM), Voronoi analysis of hexagonal cones (N%6), and regularity (REG), all of which can be evaluated in the AO Detect program. DM, SM, N%6, and REG are abbreviations employed by AO Detect. DM, expressed in [1/mm${}^{2}$], has an inverse correlation with SM, which measures the neighbor distance of each cone [μm]. The N%6 analysis is performed by dividing the number of Voronoi cells (i.e., six-sided cells) by the total number of cells, expressing the percentage of hexagonal cells in the image. REG, along with the N%6 parameter (both expressed in [%]), is crucial in excluding inaccuracies resulting from cell identification errors [48].

Patients diagnosed with CD, CRD, or STGD, confirmed previously through clinical history, eye fundus imaging, electrophysiological testing, perimetry, FAF, and, in some cases, fluorescein angiography, had their eyes included in the study. Genetic testing was performed on all patients in the Stargardt group, revealing *ABCA4* gene mutations in 12 individuals. Gene mutations leading to STGD or another IRD were not confirmed in other patients.

Patients with other ocular pathologies (such as glaucoma, cataract), those with a history of ocular surgeries or uveitis, and diabetic patients were excluded from the study. Cases where AO data collection was impossible due to poor visual acuity, nystagmus, or a lack of fixation were also excluded. This led to the exclusion of one eye each from the STGD and CD groups.

The study group included 53 eyes from 28 patients: 36 eyes (from 19 patients) with STGD, 8 eyes (from 4 patients) with CRD, and 9 eyes (from 5 patients) with CD. The control group comprised 14 healthy right eyes from 14 patients. Exclusion criteria for the control group included past ocular pathologies, increased body weight (BMI>25 kg/m${}^{2}$), systemic hypertension, and diabetes. All eyes in the control group had a BCVA of 1.0.

Table 1 presents the demographic data and BCVA of the study and control groups.

All patients provided written consent for their participation in the study, which adhered to the tenets of the Declaration of Helsinki and was approved by the bioethics commission of the Medical University of Warsaw (KB/87/2015). The exams were conducted in the Department of Ophthalmology of the Medical University of Warsaw, in the SPKSO Ophthalmic University Hospital in Warsaw.

Patients with IRDs had both eyes examined, while the control group had their right eyes examined, using the Rtx1™. Measurements were taken at four positions: $2^{\circ }$ from the foveola in the superior, inferior, temporal, and nasal quadrants.

Before each examination, the BCVA was checked using a Snellen chart, and the axial length of each eye was measured using the LS 900 (Haag Streit, Wedel, Germany). After administering one drop of topical 1% tropicamide to dilate the pupils, the Rtx1™ test was performed. After image acquisition, the images were processed with AO Detect to analyze the photoreceptor parameters DM, SM, REG, and N%6.

Data were assessed for a normal distribution using the Shapiro–Wilk test. If data were normally distributed, Student’s *t*-test for independent variables was used to compare mean values. If the data did not follow a normal distribution, the non-parametric U Mann–Whitney test was used to compare continuous variables between two groups of observations. If there were more than two groups, one-way ANOVA (parametric test) or the Kruskal–Wallis test (non-parametric) was used to compare differences, depending on the assumptions. ANOVA was followed by the HSD Tukey’s post-hoc test and the Kruskal–Wallis test was followed by Dunn’s post-hoc test. The results from both post-hoc tests were adjusted using Bonferroni correction.

A multivariate logistic model was used to examine the relationship between the existence of missing data for respective quadrants and the available variables. The model with the best fit was selected based on the Akaike Information Criterion (AIC). The exponents of the β coefficients (exp(β)) were calculated to express the unit odds ratio. When a predictor was a dichotomous categorical variable, one was assumed for the event and zero otherwise. The odds ratio represented the ratio of probability of the occurrence and non-occurrence of a given event.

In this analysis, the level of statistical significance was set to p=0.05. All calculations were conducted using R *(version 4.0.2)*.

The values of DM, SM, REG, and N%6 for both the study and control groups are presented in Table 2.

## 3. Outcomes

### 3.1. Differences in Cone Density (DM), Cone Spacing (SM), Cone Regularity (REG), and Voronoi Analysis (N%6) between the Study and Control Groups

A statistically significant difference was observed between the control group and those with IRDs concerning DM (10,111.33/mm${}^{2}$ vs. 25,646.42/mm${}^{2}$, p<0.001), SM (12.11 μm vs. 6.91 μm, p<0.001), REG (83.74% vs. 90.98%, p<0.001), and N%6 (45.21% vs. 48.43%, p=0.008) collectively across all quadrants, as detailed in Table 2. Additionally, a significant difference between the study group and the control group was noted in DM, SM, and REG for each quadrant, and in N%6 in the superior and inferior quadrants, as shown in Table 3.

### 3.2. Differences in DM, SM, REG, and N%6 between the Right Eyes of the Study Group and Controls

The analysis of data specifically from the right eyes revealed statistically significant differences between patients and the control groups in the mean values of DM, SM, and REG (p=0.003, p=0.017, p=0.035, respectively). However, there was no significant difference observed in N%6 (p=0.220) between these groups, as demonstrated in Table 4.

### 3.3. Differences in BCVA, DM, SM, and REG between Right and Left Eyes with IRDs

No statistically significant differences were observed in BCVA (p=0.218), DM (p=0.172), SM (p=0.812), or REG (p=0.156) between the right and left eyes in the study group, as evidenced in Table 5.

### 3.4. Differences in DM and SM among Eyes with CD, CRD, and STGD

The mean DM values were 8900.39/mm${}^{2}$, 9296.32/mm${}^{2}$, and 16,209.66/mm${}^{2}$ for eyes with CD, CRD, and STGD, respectively. Meanwhile, the average SM was 12.37μm for CD, 14.82μm for CRD, and 9.65μm for STGD.

In eyes with IRDs, the mean SM and DM values showed significant differences based on the diagnosis: CD, CRD, or STGD (p=0.006 and p=0.002 for DM and SM, respectively), as demonstrated in Table 6. The highest average DM was observed in eyes with Stargardt’s disease (mean DM 16,209.66/mm${}^{2}$, SD 8024.64/mm${}^{2}$), which also exhibited the lowest average SM (9.65μm, SD 2.87μm).

However, differences in REG and N%6 among the CD, CRD, and STGD groups were not statistically significant (p=0.334 and p=0.828). Furthermore, the aforementioned correlations were not statistically significant when analyzing each quadrant.

REG was significantly correlated (p=0.044) with the diagnosis of STGD, CD, or CRD in the temporal quadrant for the right eye, as depicted in Table 7. No such correlation was found in the remaining quadrants of the right eye or in any quadrant of the left eye.

Dunn’s post-hoc analysis revealed a statistically significant difference in DM and SM between CD and STGD (mean DM 8,900.39/mm${}^{2}$ (SD 3022.87/mm${}^{2}$) vs. 16,209.66/mm${}^{2}$ (SD 8024.64/mm${}^{2}$), p=0.002; mean SM 12.37μm (SD 2.96μm) vs. 9.65μm (SD 2.87μm), p=0.014).

Likewise, a statistically significant difference in DM and SM was found between CRD and STGD (mean DM 9296.32/mm${}^{2}$ (SD 2965.31/mm${}^{2}$) vs. 16,209.66/mm${}^{2}$ (SD 8024.64/mm${}^{2}$), p=0.003; mean SM 14.82μm (SD 8.28μm) vs. 9.65μm (SD 2.87μm), p=0.027).

**Figure 8:**
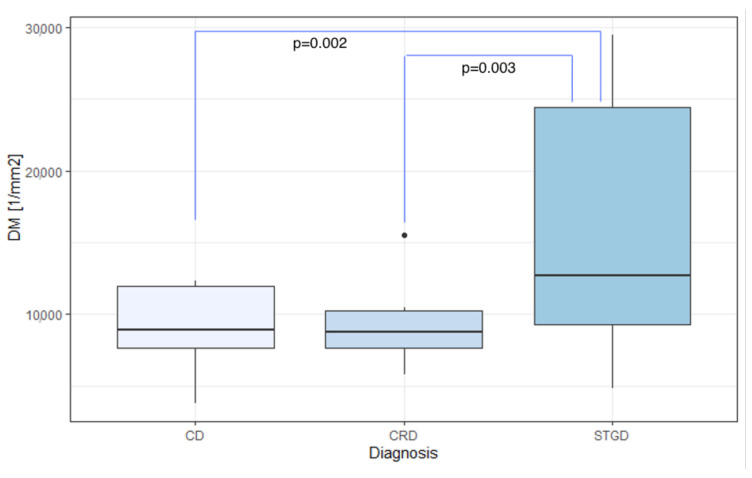
Comparison of DM among CD, CRD, and STGD groups. DM: cone density [1/mm${}^{2}$]; CD: cone dystrophy; CRD: cone-rod dystrophy; STGD: Stargardt disease.

**Figure 9:**
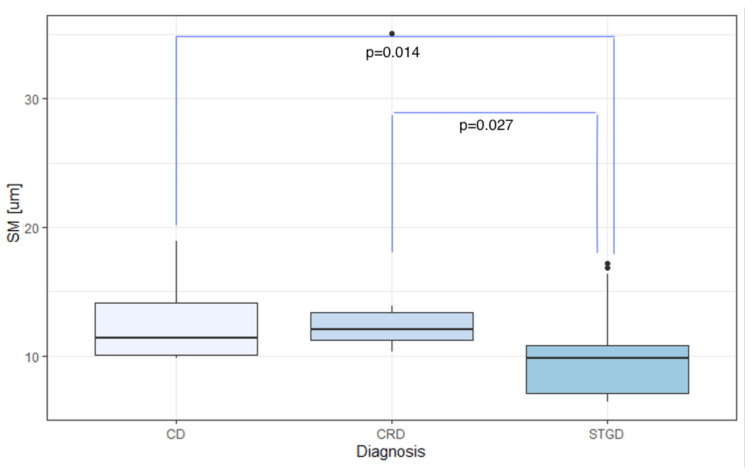
Comparison of SM among the CD, CRD, and STGD groups. SM: cone spacing [μm]; CD: cone dystrophy; CRD: cone-rod dystrophy; STGD: Stargardt disease.

The data discussed above are illustrated in Figure 8 and Figure 9.

### 3.5. Correlation between Photoreceptor Parameters and BCVA

No significant correlation was observed between BCVA and any of the measured parameters (DM, SM, REG, or N%6) in the study group, as illustrated in Table 8. Since all eyes in the control group had a BCVA of 1.0, no correlation calculations were conducted between BCVA and the AO cone parameters (DM, SM, REG, N%6) in the controls.

Similarly, no significant correlation was found between BCVA and DM, SM, and REG in the right eye (p=0.877, p=0.737, and p=0.130 for DM, SM, and REG, respectively) or in the left eye (p=0.208, p=0.106, and p=0.349 for DM, SM, and REG, respectively). These findings are shown in Table 9.

### 3.6. Correlation between Photoreceptor Parameters and Age

There was no significant correlation observed between age and the adaptive optics parameters (DM, SM, REG, and N%6) in either the eyes with IRDs or the control group, as illustrated in Table 10.

### 3.7. Correlation of DM and SM with the Probability of Incomplete Data Acquisition

During data collection, cases arose where image acquisition was impossible in all four quadrants or where the image quality was insufficient for analysis. This was mainly observed in patients with poor fixation and nystagmus. We investigated the factors that could potentially reduce the likelihood of obtaining a complete, high-quality dataset. Incomplete data were defined as the acquisition of fewer than four images suitable for analysis, while complete data entailed acquiring one image in each quadrant (superior, inferior, temporal, and nasal) suitable for analysis.

Incomplete data collection did not depend on patients’ BCVA, age, sex, or diagnosis (CD, CRD, or STGD), nor was it correlated with DM, SM, or REG, as shown in Table 11.

Nonetheless, univariate logistic regression model analysis indicated that DM was a statistically significant factor (OR=0.72, CI 0.55–0.90, p=0.008) affecting incomplete data collection in the study group. A decline in DM by 1000/mm${}^{2}$ increased the odds of incomplete data collection by 1.39 times. Owing to the substantial scale of DM, this variable was rescaled (divided by 1000) to derive a more reliable estimate of the odds ratio (OR). Age, sex, BCVA, diagnosis, SM, and regularity were not identified as significant factors in the univariate logistic regression model.

In a separate analysis of the univariate logistic regression models for the right and left eyes, SM emerged as a significant factor impacting incomplete data collection for the left eye: each unit increase in SM multiplied the odds of failure by 2.14 (p=0.019). DM was also identified as a significant factor (p=0.011), but its overall impact was negligible (OR=1).

There were no cases of incomplete data collection in the control group.

## 4. Discussion

### 4.1. Evaluation of Cones and Rods in IRDs

There exist several studies providing thorough analyses of adaptive optics (AO) imaging and numeric parameters in healthy populations. Using the Rtx1™, the mean values of the AO parameters in a healthy population are as follows: DM 19,453/mm${}^{2}$, SM 7.96μm, and N%6 46.7%, with no significant difference found between the right and left eyes [46].

The characteristics of IRDs in adaptive optics are known to differ from those of healthy eyes [14,25,26,30,37,38,57].

Wolfing et al.’s study [34], the first to describe photoreceptor image disruption in CRD, focused on imaging the retina of a single patient. This revealed an abnormal cone density with a 6.6-fold reduction compared to normative data for healthy eyes.

An investigation of three patients with CRD and their three healthy relatives using AOSLO demonstrated that the cone-to-rod spacing ratio was increased in all the CRD patients. This suggests the dominance of cone loss over rod loss in CRD. Despite the symptoms of CRD, one patient (aged 18) showed no abnormalities in cone spacing compared to normative data. Another patient (aged 12), however, displayed increased cone spacing in all measured eccentricities. Rod loss increased with eccentricity. These data suggest a polymorphic course of CRD in patients, even within the same family, and a higher degree of rod loss in more peripheral areas of the retina [38].

Sahel et al. [57] assessed AOFIO in 10 eyes of 10 patients with RCD. While changes in cone morphology were described, no numeric parameters were reported. A study by Duncan et al. [37] reported significant changes in SM between healthy retinas and those with retinitis pigmentosa (5 eyes) and cone-rod dystrophy (3 eyes). Foote et al. [14] analyzed cone spacing in 15 eyes of 14 patients with RCD and found a correlation between SM and macular sensitivity.

Chen et al. [25] reported cone abnormalities, such as increased cone spacing in regions of abnormal FAF imaging, in 12 patients with Stargardt disease. The study by Song et al. [26] reported changes not only in cone spacing but also in rod spacing in two patients with Stargardt disease. The cone density in the foveal region peaked at around 48,300/mm${}^{2}$ in one patient and was impossible to measure in the other patient since no cones were identified in the foveal region.

In another study, Song et al. [30] reported a 50% increase in cone spacing and a 30% increase in rod spacing in patients with STGD. Photoreceptor changes did not uniformly correspond with FAF abnormalities. The study included three patients with STGD compared to a healthy control. The authors highlighted abnormalities in photoreceptor appearance and the presence of dark spots (‘dark spaces’) in the mosaic.

There have been studies, including ours, that have confirmed the correlation of AO findings with functional and structural changes. Duncan et al. [37] found a significant correlation of cone spacing in patients with RP and CRD with the foveal threshold in perimetry, electrophysiological changes in mfERG, and BCVA. This study group included eight eyes with IRDs.

Another study compared AO imaging with perimetrical and electrophysiological changes, as well as contrast sensitivity. The correlation of these parameters was confirmed. The study group was heterogeneous and consisted of three patients with RCD, one patient with CRD, and one with juvenile macular dystrophy [35].

### 4.2. Early Diagnosis of IRDs

Several studies highlight the potential role of adaptive optics in the early diagnosis of retinal dystrophies. For instance, Palejwala et al. reported that cone loss measured by adaptive optics flood illumination ophthalmoscopy (AOFIO) can show signs of inherited retinal dystrophy before the symptoms of vision loss appear, as evidenced in a 7-year-old patient [58]. This patient was diagnosed with a mutation in the *ELOVL4* gene, which is responsible for autosomal dominant Stargardt-like macular dystrophy.

The case studies described by Ito et al. [59] and Kubota et al. [60] underscore the importance of assessing cone density in the diagnosis of cone dystrophy and atypical Usher syndrome.

Understanding the phenotypes of cones and rods, along with their changes during the progression of retinal diseases, is considered to be vital for future studies on inherited retinal dystrophies (IRDs) [61].

### 4.3. Potential for Future Advancement in Adaptive Optics

The loss of central vision and unstable fixation pose challenges to obtaining high-quality images. Furthermore, in IRDs, the cone spacing (SM) and cone density (DM) vary within the same eye, a factor described in the context of Stargardt disease [62]. Chen et al. [25] reported difficulties in obtaining AO images in 4 out of 12 patients with STGD.

The accumulation of lipofuscin may also lead to the aberrant acquisition of a cone mosaic appearance in certain regions. To circumvent this issue, we calculated results based on average measurements from four different regions. Other proposed solutions include generating cone density deviation maps and correlating them with OCT and microperimetry data [63]. Conversely, some studies suggest that in patients with RP, the repeatability of AO measurements is comparable to that in healthy subjects when measured with the i2K Retina device [64]. However, we found no data for maculopathies similar to the the ones investigated in our study.

Rtx1™, despite its widespread use and the existence of population data, has some limitations, such as difficulty in examining foveal cones, which are crucial in IRDs [48]. We, as with other researchers using Rtx1™, circumvented this problem by taking measurements two degrees away from the fovea in different directions.

One difficulty concerning the adaptive optics scanning laser ophthalmoscopy (AOSLO) imaging of IRDs is the possible imprecision in detecting photoreceptors in abnormal retinas [65]. Deep learning solutions are being developed to facilitate the automatic recognition of cones in IRDs, even in microscopic pathologies previously unseen [45].

The diagnostic process for IRDs is often lengthy and challenging due to the overlapping symptoms and multiple gene mutations causing each dystrophy. Artificial intelligence models have been proposed to facilitate the diagnosis of IRDs based on clinical images. The model assessment includes AO imaging of the retina, as well as fundus imaging, fluoresceine and indocyanine-green angiography, autofluorescence, and OCT. This highlights the potential role of adaptive optics as a crucial tool in diagnosing rare retinal diseases [29,66,67].

AO imaging, however, has universal limitations: it does not assess foveal cones or rods and is a costly solution. The need for the precise visualization of the photoreceptor mosaics has spurred the development of other imaging techniques. For instance, a study comparing AOSLO images with those from the Heidelberg Engineering SPECTRALIS High Magnification Module (HMM) in Stargardt disease showed promising quality and potential for HMM development [48].

### 4.4. The Research Group

Our study underscores significant differences in cone parameters, specifically in cone density (DM) and cone spacing (SM), between STGD, CD, and CRD. These findings could potentially streamline the differential diagnosis among IRDs that affect the macula.

The molecular etiology of STGD differs from that of CD and CRD, primarily impacting the retinal pigment epithelium (RPE) instead of the photoreceptors. In our study, the DM and SM parameters in the STGD group demonstrated the highest standard deviations among all groups.

A limitation of our study is the absence of a confirmed genetic etiology of IRD in all participants.

In several other studies [34,37], the genetic mutations causing CRD or RP were not reported for the majority of the patients examined. Similarly, more recent reports [14,57] do not provide precise information on mutations in all subjects.

Since the clinical symptoms of IRDs can overlap, diagnosis without confirming the genetic mutation may be questionable. There is a need for studies focusing on the differences in photoreceptor changes, as seen in AO, between eyes with varying genetic causes of retinal degeneration.

Studying rare conditions such as IRDs is challenging due to the difficulty in gathering a sufficiently large research group for reliable statistical analysis. However, we mitigated this issue by successfully assembling a large study group and comparing their data with outcomes from healthy retinas.

Ratnam et al. [55] described foveal changes in 18 eyes from 18 patients diagnosed with various types of IRDs. Tuten et al. [68] examined 12 eyes from 12 patients diagnosed with choroideremia using AO-SLO and AO-microperimetry. To the best of our knowledge, our study represents the largest research group in terms of AO imaging in IRDs.

### 4.5. Longitudinal Observation

Adaptive optics (AO) is a relatively recent advancement in the field of ophthalmic imaging. As such, there are still few studies examining changes over time. Roshandel et al. [69] conducted a six-month observation study on photoreceptor changes, confirming significant parafoveal cone loss over the observation period in rod-cone dystrophy (RCD). Foote et al. [14] documented an increase in cone spacing in 15 eyes of 14 RCD patients over a three-year observation period. Furthermore, Chen et al. [25] described the longitudinal progression of photoreceptor changes in two Stargardt disease patients over 27 months. However, we found no such studies for cone-rod dystrophy or cone dystrophy.

Ziccardi et al. explored the possibility of monitoring the progression of retinitis pigmentosa (RP) associated with a mutation in the *RP1L1* gene in three patients [70]. Over a two-year follow-up, they noted a significant reduction in DM in the proband, with the most substantial reduction observed 2${}^{\circ }$ from the fovea.

Other retinal conditions, such as retinal detachment, are also subjects of investigation for photoreceptor damage. Potic et al. [71] used AO to examine visual acuity and cone density in eyes after retinal detachment repair, with a follow-up time of three months.

## 5. Conclusions

Our study confirmed that photoreceptor parameters in eyes with IRDs distinctly diverge from those in healthy eyes. An examination utilizing adaptive optics could potentially facilitate the differentiation between STGD and CD, as well as between STGD and CRD.

We advocate for more studies focused on the adaptive optics parameters unique to each genetic mutation. These research efforts could yield fresh insights into the etiology of photoreceptor degeneration in varying conditions.

From our perspective, there exists a significant demand for longitudinal studies that assess the progression of photoreceptor changes over extended periods.

## Figures and Tables

**Table 1:** Demographic data, diagnosis, eye laterality (left/right), and BCVA for both the study group and the control group. BCVA: best-corrected visual acuity; CD: cone dystrophy; CRD: cone-rod dystrophy; STGD: Stargardt disease.

	Study Group (N=53)	Control Group (N=14)
Age		
Mean (SD)	44.02(14.24)	46.93(9.28)
Median (IQR)	44 (35–54)	47.5 (42.25–55.25)
Range	19–73	31–59
Sex		
Female	60.4%(N=32)	92.9%(N=13)
Male	39.6%(N=21)	7.1%(N=1)
Diagnosis		
CD	17%(N=9)	-
CRD	15.1%(N=8)	-
STGD	67.9%(N=36)	-
Eye		
Right	28	14
Left	25	0
BCVA		
Mean (SD)	0.13(0.14)	1.0
Median (IQR)	0.07 (0.05–0.16)	1.0
Range	0.01–0.7	1.0

**Table 2:** Comparison of DM, SM, REG, and N%6 between the study and control groups. DM: cone density [1/mm${}^{2}$]; SM: cone spacing [μm]; REG: cone regularity [%]; N%6: Voronoi analysis [%]. The bold was used in all *p*-Values lower than 0.05 (=with statistical significance).

	Study Group (N=53)	Control Group (N=14)	*p*-Value (U Mann–Whitney)
DM			<0.001
Mean (SD)	10,111.33 (3198.77)	25,656.42 (2132.93)	
Median (IQR)	10,228.25 (7943.67–12,341.25)	24,961.54 (24,046.79–27,320.94)	
Range	3830–16,341.25	22,977.75–29,455.25	
SM			<0.001
Mean (SD)	12.11(4.17)	6.91(0.29)	
Median (IQR)	10.91 (9.92–12.24)	7 (6.68–7.13)	
Range	8.59–35.08	6.42–7.3	
REG			<0.001
Mean (SD)	83.74(8.54)	90.98(1.8)	
Median (IQR)	86.09 (80.81–88.96)	91.25 (89.64–92.18)	
Range	48.28–96.77	87.81–94.07	
N%6			0.008
Mean (SD)	45.21(8.21)	48.43(3.25)	
Median (IQR)	43.5 (40.5–48)	48.88 (48.18–49.58)	
Range	27.65–73.75	41.8–53.27	

**Table 3:** Comparison between the control and study groups, with evaluations made in each quadrant: temporal, nasal, superior, and inferior. DM_T: cone density in the temporal quadrant; SM_T: cone spacing in the temporal quadrant; REG_T: cone regularity in the temporal quadrant; N%6_T: Voronoi analysis in the temporal quadrant; DM_N: cone density in the nasal quadrant; SM_N: cone spacing in the nasal quadrant; REG_N: cone regularity in the nasal quadrant; N%6_N: Voronoi analysis in the nasal quadrant; DM_S: cone density in the superior quadrant; SM_S: cone spacing in the superior quadrant; REG_S: cone regularity in the superior quadrant; N%6_S: Voronoi analysis in the superior quadrant; DM_I: cone density in the inferior quadrant; SM_I: cone spacing in the inferior quadrant; REG_I: cone regularity in the inferior quadrant; N%6_I: Voronoi analysis in the inferior quadrant. The bold was used in all *p*-Values lower than 0.05 (=with statistical significance).

	Study Group (N=53)	Control Group (N=14)	*p*-Value (U Mann–Whitney)
Mean DM [1/mm${}^{2}$] (SD)			
DM_T	10,893.92 (6038.18)	26,729.98 (2058.61)	<0.001
DM_N	9718.94(4263.11)	25,585.69 (2153.57)	<0.001
DM_S	9673.34(3648.9)	25,386.9 (2768.69)	<0.001
DM_I	10,159.14 (4408.24)	24,923.12 (3023.91)	<0.001
Mean SM [μm] (SD)			
SM_T	11.32(3.07)	6.76(0.27)	<0.001
SM_N	11.82(3.71)	6.91(0.3)	<0.001
SM_S	11.8(3.39)	6.94(0.37)	<0.001
SM_I	13.52(13.13)	7.03(0.42)	<0.001
Mean REG [%] (SD)			<0.001
REG_T	81.37(15)	94.11(3.2)	<0.001
REG_N	85.44(11.41)	94.48(2.58)	0.001
REG_S	82.41(14.18)	81.05(3.6)	0.029
REG_I	85.53(10.25)	94.28(3.18)	<0.001
Mean N%6 [%] (SD)			
N%6_T	43.02(9.05)	48.02(6.03)	0.061
N%6_N	47.04(15.39)	47.41(3.96)	0.162
N%6_S	45.88(13.24)	50.73(6.11)	0.036
N%6_I	44.91(14.09)	47.56(5.27)	0.031

**Table 4:** Comparison of the mean values of DM, SM, REG, and N%6 between the study group and the control group for the right eye. DM: cone density [1/mm${}^{2}$]; SM: cone spacing [μm]; REG: cone regularity [%]; N%6: Voronoi analysis [%]. The bold was used in all *p*-Values lower than 0.05 (=with statistical significance).

	Study Group (N=29)	Control Group (N=14)	*p*-Value (U Mann–Whitney)
DM	10,154.52 (3641.81)	25,656.42 (2132.93)	0.003
SM	11.64(2.83)	6.91(0.29)	0.017
REG	82.69(8.32)	90.98(1.8)	0.035
N%6	45.2(10.35)	48.43(3.25)	0.220

**Table 5:** Characteristics of BCVA, DM, SM, REG in right and left eyes with IRDs. BCVA: best-corrected visual acuity; DM: cone density [1/mm${}^{2}$]; SM: cone spacing [μm]; REG: cone regularity [%]; IRDs: inherited retinal dystrophies.

	Right Eye (N=28)	Left Eye (N=25)	*p*-Value (Test)
BCVA			
Mean (SD)	0.14(0.17)	0.11(0.16)	0.218 (*t*-test)
Median (IQR)	0.06 (0.04–0.2)	0.05 (0.04–0.12)	
Range	0.01–0.8	0.01–0.8	
DM			
Mean (SD)	10,357.02(3246.84)	9767.12(3224.25)	0.172 (*t*-test)
Median (IQR)	9396.5 (8420.12–12,993.88)	10,480.5 (6807–12,074.25)	
Range	3830–15,499.88	4584.33–16,341.25	
SM			
Mean (SD)	12.4(5.13)	11.9(2.95)	0.812 (Wilcoxon)
Median (IQR)	11.31 (9.91–12.11)	10.53 (10.06–13.21)	
Range	8.85–35.08	8.59–21.18	
REG			
Mean (SD)	83.25(7.53)	85.57(6.21)	0.156 (Wilcoxon)
Median (IQR)	85.66 (78.39–88.39)	86.17 (84.48–88.96)	
Range	60.66–96.77	66.67–95.84	

**Table 6:** Comparison of mean DM, SM, REG, and N%6 values among eyes with CD, CRD, and STGD. DM: cone density [1/mm${}^{2}$]; SM: cone spacing [μm]; REG: cone regularity [%]; N%6: Voronoi analysis [%]; CD: cone dystrophy; CRD: cone-rod dystrophy; STGD: Stargardt disease. The bold was used in all *p*-Values lower than 0.05 (=with statistical significance).

	CD (N=9)	CRD (N=8)	STGD (N=36)	*p*-Value (Kruskal–Wallis)
DM	8900.39(3022.87)	9296.32(2965.31)	16,209.66 (8024.64)	0.006
SM	12.37(2.96)	14.82(8.28)	9.65(2.87)	0.002
REG	87.22(4.98)	82(10.31)	83.47(10.82)	0.334
N%6	46.18(5.4)	45.43(12.89)	46.29(9.38)	0.828

**Table 7:** Correlation of REG in each quadrant with the diagnosis for right eye. REG_T [%]: cone regularity in temporal quadrant; REG_N [%]: cone regularity in nasal quadrant; REG_S [%]: cone regularity in superior quadrant; REG_I [%]: cone regularity in inferior quadrant. CD: cone dystrophy; CRD: cone-rod dystrophy; STGD: Stargardt disease. The bold was used in all *p*-Values lower than 0.05 (=with statistical significance).

	CD (N=5)	CRD (N=4)	STGD (N=19)	*p*-Value (Kruskal–Wallis)
REG_T				0.044
Mean (SD)	0.91 (0.05)	0.89 (0.05)	0.76 (0.2)	
Median (IQR)	0.92 (0.86–0.95)	0.91 (0.88–0.92)	0.85 (0.75–0.88)	
Range	0.84–0.96	0.82–0.94	0.33–0.94	
REG_N				0.953
Mean (SD)	0.85 (0.12)	0.86 (0.04)	0.87 (0.08)	
Median (IQR)	0.85 (0.84–0.87)	0.87 (0.85–0.89)	0.85 (0.81–0.92)	
Range	0.67–1	0.8–0.89	0.75–1	
REG_S				0.681
Mean (SD)	0.87 (0.07)	0.8 (0.14)	0.81 (0.14)	
Median (IQR)	0.87 (0.86–0.89)	0.85 (0.76–0.89)	0.87 (0.74–0.88)	
Range	0.77–0.96	0.6–0.9	0.5–1	
REG_I				0.511
Mean (SD)	0.87 (0.04)	0.87 (0.03)	0.85 (0.07)	
Median (IQR)	0.89 (0.86–0.89)	0.88 (0.86–0.89)	0.85 (0.81–0.88)	
Range	0.8–0.9	0.82–0.9	0.71–1	

**Table 8:** Spearman’s correlation coefficients between BVCA and the mean values of DM, SM, REG, and N%6 in the eyes with IRDs. DM: cone density [1/mm${}^{2}$]; SM: cone spacing [μm]; REG: cone regularity [%]; N%6: Voronoi analysis [%].

	Correlation Coefficient (r)	*p*-Value
DM	0.07	0.612
SM	−0.109	0.436
REG	0.191	0.170
N%6	0.013	0.924

**Table 9:** Spearman’s correlation coefficients between BCVA and DM, SM, and REG in the right eye and the left eye with IRDs. DM: cone density [1/mm${}^{2}$]; IRDs: inherited retinal dystrophies; SM: cone spacing [μm]; REG: cone regularity [%]; N%6: Voronoi analysis [%].

	Correlation Coefficient (r)	*p*-Value
Right eyes		
DM	0.031	0.877
SM	−0.068	0.737
REG	0.299	0.13
Left eyes		
DM	0.261	0.208
SM	−0.331	0.106
REG	0.196	0.349

**Table 10:** Spearman’s correlation coefficients between age and DM, SM, REG, and N%6 in eyes with IRDs and in healthy eyes. DM: cone density [1/mm${}^{2}$]; IRDs: inherited retinal dystrophies; SM: cone spacing [μm]; REG: cone regularity [%]; N%6: Voronoi analysis [%].

	Correlation Coefficient (r)	*p*-Value
Study group		
DM	−0.146	0.295
SM	0.186	0.184
REG	−0.152	0.277
N%6	0.075	0.593
Control group		
DM	−0.286	0.321
SM	0.299	0.3
REG	0.133	0.65
N%6	0.325	0.257

**Table 11:** Descriptive characteristics concerning complete or incomplete data (with complete data indicating that 4 measurements provided an image suitable for analysis). BCVA: best-corrected visual acuity; DM: cone density [1/mm${}^{2}$]; SM: cone spacing [μm]; REG: cone regularity [%]; CD: cone dystrophy; CRD: cone-rod dystrophy; STGD: Stargardt disease.

		Incomplete Data (N=12)	Complete Data (N=41)	*p*-Value *(Test)*
Mean age (SD)		45.63(14.3)	41.92(13.95)	0.360
				(*t*-test)
Sex				
	Male	50%(N=6)	36.6%(N=15)	0.839
	Female	50%(N=6)	63.4%(N=26)	(chi-squared)
Diagnosis				
	CD	25.0%(N=3)	14.6%(N=6)	0.838
	CRD	16.7%(N=2)	14.6%(N=6)	(Fisher)
	STGD	58.3%(N=7)	70.7%(N=29)	
BCVA				
	Mean (SD)	0.1 (0.12)	0.15 (0.19)	0.309
	Median (IQR)	0.05 (0.04–0.11)	0.1 (0.04–0.2)	(U Mann–Whitney)
	Range	0.01–0.4	0.01–0.8	
DM				
	Mean (SD)	9667.5 (3092.92)	10,673.83 (3263.13)	0.284
	Median (IQR)	9180 (8083–10,990.75)	10,228.25 (8593–13,400.5)	(U Mann–Whitney)
	Range	5292.75–15,499.88	3830–15,499.88	
SM				
	Mean (SD)	13.28 (5.93)	11.99 (4.67)	0.103
	Median (IQR)	11.39 (10.91–13.89)	10.91 (9.76–11.99)	(U Mann–Whitney)
	Range	9.04–35.08	8.85–35.08	
REG				
	Mean (SD)	84.19 (7.3)	82.82 (7.59)	0.889
	Median (IQR)	85.66 (77.46–88.31)	85.66 (79.05–88.47)	(U Mann–Whitney)
	Range	72.81–96.77	60.66–91.31	

## Data Availability

Not applicable.

## Abbreviations

The following abbreviations are used in this manuscript:

AMD	Age-related macular degeneration
AO	Adaptive optics
AOFIO	Adaptive optics flood illuminated ophthalmoscope
AOSLO	Adaptive optics scanning laser ophthalmoscopy
CD	Cone dystrophy
CDSR	Cone dystrophy with supernormal rod electroretinogram
CRD	Cone-rod dystrophy
BCVA	Best-corrected visual acuity
MDPI	Multidisciplinary Digital Publishing Institute
DOAJ	Directory of Open Access Journals
DM	Cone density [1/mm${}^{2}$]
FAF	Fundus autofluorescence
FFA	Fluorescein angiography
HMM	High Magnification Module
IQR	Interquartile range
IRD	Inherited retinal dystrophy
LCA	Leber congenital amaurosis
mfERG	Multifocal electroretinography
N%6	Voronoi analysis of hexagonal cones [%]
RCD	Rod-cone dystrophy
REG	Cone regularity [%]
RNFL	Retinal nerve fiber layer
RP	Retinitis pigmentosa
RPE	Retinal pigment epithelium
SD	Standard deviation
SD-OCT	Spectral-domain optical coherent tomography
SM	Cone spacing [μm]
STGD	Stargardt disease
OR	Odds ratio
